# Thrombosis of pancreatic arteriovenous malformation induced by diagnostic angiography: case report

**DOI:** 10.1186/s12876-016-0485-5

**Published:** 2016-07-11

**Authors:** Jernej Vidmar, Mirko Omejc, Rok Dežman, Peter Popovič

**Affiliations:** Institute of Physiology, Medical Faculty, University of Ljubljana, Zaloska cesta 4, 1000 Ljubljana, Slovenia; Jozef Stefan Institute, Laboratory of Magnetic Resonance Imaging, Ljubljana, Slovenia; Clinical Department of Abdominal Surgery, University Medical Centre, Ljubljana, Slovenia; Institute of Radiology, University Medical Centre, Ljubljana, Slovenia

**Keywords:** Pancreatic arteriovenous malformation, Diagnostic angiography, Abdominal MRI, MR cholangiopancreatography, Acute pancreatitis

## Abstract

**Background:**

We report on a case of pancreatic arteriovenous malformation (PAVM) that obliterated shortly after diagnostic angiography (DSA). PAVM is a rare anomaly that presents with upper abdominal pain, signs of acute pancreatitis and massive gastrointestinal bleeding. The management of PAVM is rather complex, with complete treatment usually accomplished only by a total extirpation of the affected organ or at least its involved portion. DSA prior to treatment decisions is helpful for characterizing symptomatic PAVM, since it can clearly depict the related vascular networks. In addition, interventional therapy can be performed immediately after diagnosis.

**Case presentation:**

A 39-old male was admitted due to recurring upper abdominal pain that lasted several weeks. Initial examination revealed the absence of fever or jaundice, and the laboratory tests, including that for pancreatic enzymes, were unremarkable. An abdominal ultrasound (US) showed morphological and Doppler anomalies in the pancreas that were consistent with a vascular formation. A subsequent DSA depicted a medium-sized nidus, receiving blood supply from multiple origins but with no dominant artery. Coil embolization was not possible due to the small caliber of the feeding vessels. In addition, sclerotherapy was not performed so as to avoid an unnecessary wash out to the non-targeted duodenum. Consequently, the patient received no specific treatment for his symptomatic PAVM. A large increase in pancreatic enzymes was noticed shortly after the DSA procedure. Imaging follow-up by means of CT and MRI showed small amounts of peripancreatic fluid along with a limited area of intra-parenchymal necrosis, indicating necrotizing pancreatitis. In the post-angiography follow-up the patient was hemodynamically stable the entire time and was treated conservatively. The symptoms of pancreatitis improved over a few days, and the laboratory findings returned to normal ranges. Long-term follow-up by way of a contrast-enhanced CT revealed no recanalization of the thrombosed PAVM.

**Conclusion:**

The factors associated with the obliteration of PAVM during or after DSA are poorly understood. In our case it may be attributed to the low flow dynamics of PAVM, as well as to the local administration of a contrast agent. Asymptomatic PAVM, as diagnosed with non-invasive imaging techniques, should not be evaluated with DSA due to the potential risk of severe complications, such as acute pancreatitis.

## Background

Pancreatic arteriovenous malformation is a rare anomaly, which can be congenital or acquired by way of inflammation, tumor or trauma. Most patients with PAVM remain asymptomatic and have progressive portal hypertension in the absence of primary liver pathology. Symptomatic PAVM commonly presents with upper abdominal pain, signs of acute pancreatitis and gastrointestinal bleeding [1, 2]. The most frequently involved portion of the pancreas is reported to be the head (up to 60 %), followed by the body and tail, and the least frequent, the entire pancreas [3].

Treatment of PAVM is indicated only in symptomatic patients, who are the minority. The management of symptomatic PAVM is complex and involves a multidisciplinary approach with complete treatment usually accomplished only by a total extirpation of the affected organ or at least the portion involved. Although radical pancreatic surgery treats PAVM, it usually significantly affects the metabolic characteristics of the radically or totally pancreatectomized patient, in whom persistent malabsorption, deficiencies in fat-soluble vitamin and pancreatogenic diabetes are observed. Post-operative adverse chronic sequelae may also occur, including liver disease with unusual frequency, characterized by accelerated fatty infiltration and osteopenia, along with a reduction in radial bone mineral content in pancreatectomized patients within no more than 5 years after surgery [4].

In recent years, transcatheter arterial embolization (TAE), an alternative to surgical treatment, has been performed as a conservative approach. Its use is rather limited due to the etiopathogenesis of PAVM as well as the adverse effects of embolization procedures [5, 6]. Diagnostic angiography (DSA) conducted prior to treatment options serves as the gold standard in the characterization of PAVM, since it can clearly depict feeding arteries, any racemose intrapancreatic vascular network (followed by way of a transient dense pancreatic stain), early venous filling of the portal vein, and early disappearance of the pancreatic stain [7].

In this paper we report a case of PAVM in a patient who underwent DSA with subsequent obliteration and thrombosis of the lesion and resulting acute pancreatitis.

## Case presentation

A 39-old male was admitted to our hospital due to several weeks of recurring upper abdominal pain. Initial examination revealed the absence of fever or jaundice, and the patient reported no major involuntary weight loss. No relevant family or past disease history or bleeding tendency was found. The initial laboratory tests, including that for pancreatic enzymes, were unremarkable. An esophagogastroduodenoscopy, performed few days earlier, revealed small mucosal breaks consistent with grade A esophagitis according to the Los Angeles Classification [8].

### Diagnostic imaging

**Fig. 1:**
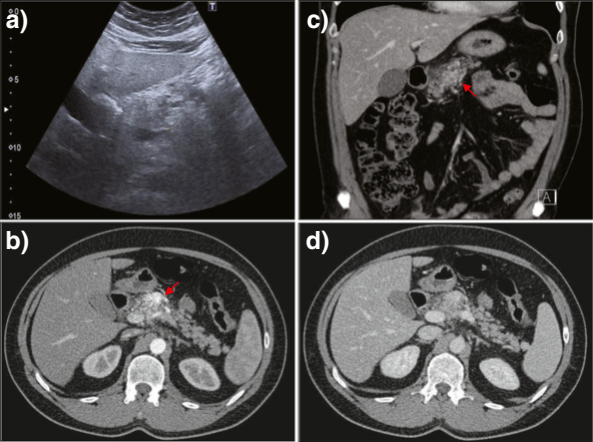
US imaging showed a structurally heterogeneous and thickened neck of the pancreas, hardly differentiated from the surrounding fat and without any dilatation of the main pancreatic duct (**a**). Contrast-enhanced CT examination through the pancreas demonstrated a mosaic hypervascular area at the same site as the initial US (**b**–**d**, *red arrows*)

B-mode ultrasound (US) showed a structurally heterogeneous and thickened neck of the pancreas, which was poorly differentiated from the surrounding fat (Fig. 1a). No dilatation of the main pancreatic duct was found. Color Doppler US demonstrated an intensive mosaic appearance in the hypoechogenic area of the lesion, and spectral Doppler analysis disclosed arterial and venous blood flow signals within the lesion. Morphological changes along with the Doppler pattern found at the initial US examination indicated a possible ongoing vascular formation within the pancreas. Subsequently, computed tomography (CT) confirmed a medium-sized (up to 4 cm) formation in the neck of the pancreas. The formation showed arterial (Fig. 1b, red arrow) and venous (Fig. 1c and d) serpinginous enhancement consistent with pancreatic vascular malformation (PAVM). A multidisciplinary team, including a surgeon, an interventional radiologist and a radiotherapist, considered various therapeutic approaches. As an alternative to a pancreaticoduodenectomy and radiotherapy, a minimally invasive treatment based on percutaneous embolization was proposed to and accepted by the patient. A CT scan was post-processed to obtain a clear vascular map of the arterial and venous pancreatic supply.

### Diagnostic angiography

Vascular access was obtained by a retrograde puncture of the femoral artery along with the introduction of a 5Fr access sheath. Selective catheterization and angiography of the celiac trunk and the superior mesenteric artery were performed by using a 5Fr Simmons sidewinder catheter Sim 1 (Cordis, Johnson & Johnsons, Miami, FL, USA). Superselective catheterization and angiography of the feeder arteries’ microcatheter were performed by a Progreat 2.4Fr (Terumo, Japan) with 0.016˝ Radifocus Guide wire GT (Terumo, Japan). The non-ionic, iso-osmolar contrast agent iodixanol 320 mg/ml (Visipaque, GE Healthcare, Chalfont St. Giles, UK) was used for the angiography in the total amount of 100 ml. The selective angiography of the celiac trunk and the superior mesenteric artery were performed with 20 ml at 5 ml/s, while super-selective angiography of feeder arteries was performed with 5–10 ml at 1 ml/s.

**Fig. 2:**
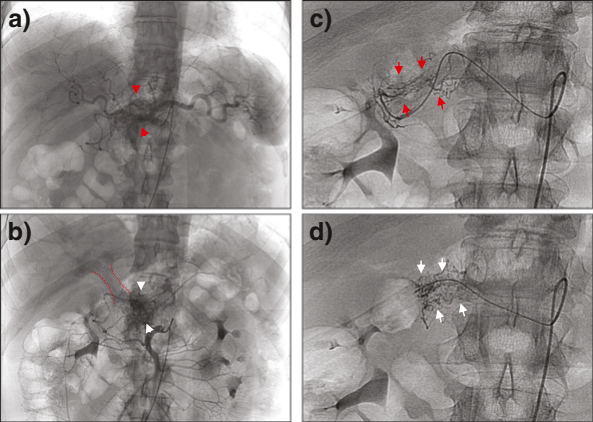
Celiacography and the selective angiography of the upper mesenteric, revealing feeding arteries from a common hepatic artery (**a**, *red arrowheads*) and from the initial portion of the upper mesenteric artery (**b**, white *arrowheads*), with early visualization of the portal vein in the same phase (*encircled in red*). Additional vascular network branching from the watershed of the gastroduodenal artery (**c**, *red arrows*) and hepatic artery proper (**d**, *white arrows*) is also shown

The celiacography and selective angiography of the upper mesenteric artery revealed that the PAVM located in the neck of the pancreas was receiving arterial blood supply from four origins: a medium-sized feeding artery branching from a common hepatic artery (Fig. 2a, red arrowheads); multiple small feeding arteries branching from the initial portion of the upper mesenteric artery (Fig. 2b, white arrowheads) with immediate opacification of the portal vein, (encircled in red); a racemose vascular network from the watershed of the gastroduodenal artery (Fig. 2c, red arrows); and the hepatic artery proper (Fig. 2d, white arrows). No dominant feeding artery was recognized among the four origins. All feeding arteries were assessed as small caliber vessels (up to 1 mm), thus TAE using spirals was not possible.

### Post-angiography follow-up

**Fig. 3:**
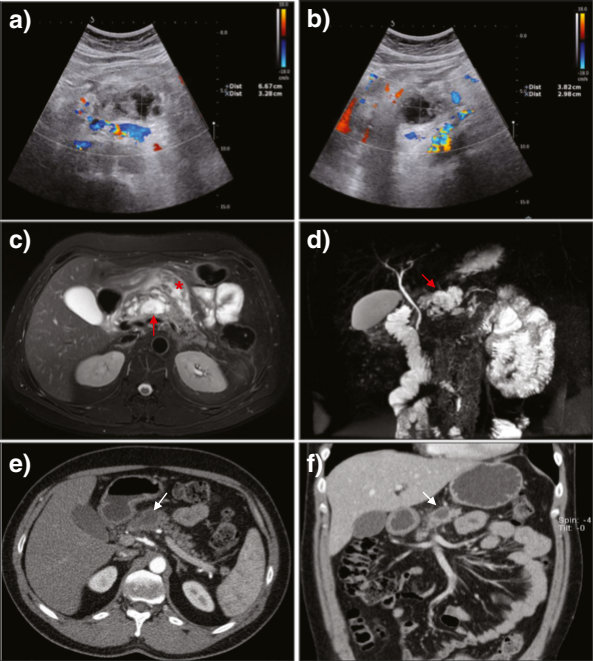
US after the diagnostic angiography revealed a progressive decrease of the serpinginous formations as well as a cystic lesion gradually increasing in size (**a**, **b**). Fat-suppressed *T*_2_-weighted MR images depicted fluid characteristics in the neck of the pancreas (**c**, *red arrow*) and in the peripancreatic fat (**c**, *red asterisk*) indicating an area of intra-parenchymal necrosis along with some peripancreatic edema. MR cholangiopancreatography confirmed minor peripancreatic fluid collection in the neck of the pancreas (**d**, *red arrow*). Long-term CT follow-up showed no evidence of previous vascular malformation along with the remaining peripancreatic fluid collection (**e**, **f**, *white arrows*)

Among the laboratory tests performed in the 24 h following the diagnostic angiography, that for pancreatic enzymes showed a sudden intensive increase above the normal range: up to 15-fold for amylase and 40-fold for lipase. Other laboratory tests, including tumor markers such as CEA, CA19-9 and chromogranin, were unremarkable. The initial US follow-up was performed three days after DSA and revealed a reduction in the serpinginous areas along with a decrease in blood flow signals (Fig. 3a and b). Follow-up employing an abdominal MRI also depicted similar serpinginous formations located in the neck of the pancreas (Fig. 3c, red arrow). The formations displayed MR characteristics of fluid (Fig. 3c) along with no flow voids and were consequently characterized as the fluid remnants of the thrombosed PAVM or differentially, a demarcated area of intraparenchymal necrosis having already occurred. The larger area of fluid remnants near the pancreas (Fig. 3c, red arrow) was characterized as a peripancreatic fluid collection, subsequently also detected by MR cholangiopancreatography (Fig. 3d, red arrow). Besides intrapancreatic changes, areas of hyperintensive signal were also found on MR images of the peripancreatic fat (Fig. 3c, red asterix), consistent with small amounts of peripancreatic fluid and edema.

Over a few days after the imaging follow-up, the patient’s symptoms dramatically improved, and laboratory findings returned to normal ranges. Several consecutive US examinations were parallel to those findings, displaying a complete resolution of the Doppler flow signal pattern in the area of pancreatic AVM as well as a small, stable peripancreatic fluid collection. Long-term follow-up by way of a contrast-enhanced CT performed three months after DSA revealed no recanalization of the thrombosed PAVM and a remaining small, stable peripancreatic fluid collection (Fig. 3e and f, white arrows).

## Discussion

Vascular malformations of the gastrointestinal tract are rather uncommon, however, when present they are predominantly found in the liver or in extrahepatic locations; there is less than 1 % incidence within the pancreas [9]. In most cases the condition is congenital and found accidentally by diagnostic imaging performed for a different reason. In the present case the PAVM was thought to be congenital due to the absence of acquired factors, such as neoplasm or trauma, and also the negative family history and lack of any vascular dysplastic disease symptoms. The patient’s primary symptom of recurrent abdominal pain could be attributed to focal ischemia, since neither bleeding from the PAVM into the pancreatic duct, nor dilatation of the duct was observed.

The standard treatment for PAVM frequently involves surgery, conservative therapy (embolization, sclerotherapy or portal venous shunt), or no treatment at all [10–12]. Surgical pancreatectomy is still considered to be the sole radical treatment for PAVM despite huge metabolic consequences sustained by the radically or totally pancreatectomized patient [13]. However, as the malformation grows, its abundant vessel network, including feeding arteries and draining veins, often increases the difficulty of surgical haemostatic treatment. Therefore, DSA prior to treatment decisions is helpful in developing the tactics of treatment, even if a diagnosis has been established by non-invasive imaging modalities [7]. In addition, subsequent interventional therapy, i.e. TAE, can be performed after the diagnosis of PAVM by this procedure [14].

In the presented case, the PAVM, characterized by way of DSA, was relatively small in size, however, branching from numerous small-caliber feeding arterioles. No dominant arteries were found; therefore, coil embolization was not possible. Additionally, TAE by means of ethylene-vinyl alcohol copolymer (Onyx, EV3, USA) was discussed as the only treatment option. In keeping with a conservative treatment approach, it was not performed so as to avoid any undesired wash out of the infused agent to the non-targeted duodenum, which could possibly cause necrosis. Sclerotherapy was also not performed, since we lacked precise initial diagnostic imaging of the vascular malformation, which would have allowed a characterization of its flow dynamics, i.e. high-flow from low-flow. In the end, the patient received no specific treatment at all.

Laboratory testing as well as imaging follow-up studies suggested that an obliteration of the PAVM appeared shortly after DSA and was followed by acute pancreatitis in accord with diagnostic criteria [15]. Several possible mechanisms that can result in the obliteration of PAVM should be considered,. The common belief is that DSA is prone to complications due to manipulation with catheters and guide wires, which are necessary for selective and super-selective studies. The most common complication of selective catheterization is a direct intimal injury, the incidence of which significantly increases with progressively super-selective catheterization [16]. Additionally, vascular spasms can occur as a result of a normal response of vascular contractile elements to the stimulation of the wall by the catheter tip, guide wire or contrast jet [17]. Although usually inconsequential, severe spasm could result in stasis and subsequent thrombosis. In the last 10 years, the incidence of intimal injury in DSA procedures has been significantly minimized by newer manipulation techniques of guide wires and catheters, as well as by the use of flexible-tipped wires and end-hole-only catheters [18]. Specifically, in our case we did not detect any delineated subintimal accumulations of contrast material within or adjacent to the opacified vascular outline, such accumulations being a consequence of direct damage to the vascular wall by the catheter tip, catheter recoil or contrast jet. Furthermore, the super-selective angiography in our case was prudently used at all times, and a minimal number of contrast injections was implemented. All catheterizations were performed at a slow speed along with relatively small quantities of contrast agents, i.e. 5–10 ml. Nevertheless, we allowed the possibility that minor intimal injury or vascular spasm could occur distally in the small caliber vessels of the PAVM, which would be undetectable by us. Another possible explanation for the obliteration of the PAVM during or shortly after DSA could involve the properties of the contrast agent and its local administration [19]. According to experimental and animal studies, even a small amount of a viscous contrast agent alters blood rheology [20, 21]. Our case could be consistent with this finding in a similar way. Local administration of a contrast agent could affect blood rheology in the small low-flow draining vessels by progressively slowing down flow dynamics and causing an occlusion of PAVM. This might further lead to an outflow obstruction of the PAVM and finally to a thrombosis of the lesion. A relatively small nidus, which was characterized in our case, could also predispose to this phenomenon.

In our case, DSA was most likely responsible for the obliteration of the PAVM, however, it also induced pancreatitis, evidenced by subsequent laboratory and imaging findings. A diagnosis of acute pancreatitis is usually made on the basis of clinical and laboratory findings, therefore our follow-up imaging (US and MRI) was primarily used to monitor possible early complications, e.g. revascularization of the thrombosed PAVM. However, clinically this phenomenon seems to be exceedingly rare. In the post-angiography follow-up the patient received no additional treatment for the symptomatic PAVM. The patient was hemodynamically stable at all times and was treated conservatively. The emphasis of our conservative treatment was on supportive measures and on the prevention of the infection of necrosis and other complications. Supportive nonsurgical management was indicated mainly because of the improved symptoms of pancreatitis over a few days and the fact that laboratory findings returned to normal ranges.

The widespread use of imaging techniques seems to be the exact reason for the increased number of reported PAVM cases in recent years. Besides PAVM detection, discrimination of flow characteristics of vascular malformations is also thought to play an essential role in determining appropriate treatment [22], i.e. surgical resection, TAE, or sclerotherapy. However, if CT is used, artery–lesion enhancement time does not reflect the hemodynamics of vascular lesions directly, as was seen in our case [23]. The two non-invasive imaging techniques that are more appropriate for the examination of vascular malformations are US and MRI [24, 25]. US has been advocated as useful for examining vascular malformations, especially differentiating low- from high-flow vascular malformations [26]. Nevertheless, US does have some limitations, including a small field of view and a restricted depth of penetration, especially with high-frequency transducers. Thus, US cannot be substituted for MRI when a determination of the full extent of vascular malformations is necessary. On the contrary, dynamic MRI enables discrimination between high-flow and low-flow vascular malformations, especially when the contrast rise time of the lesion is measured [23]. Hence, in order to improve treatment decisions, we suggest the utilization of dynamic MRI for the discrimination of high-flow from low-flow PAVM as an alternative to DSA.

## Conclusion

Diagnostic angiography prior to treatment decisions is beneficial for patients with symptomatic PAVM, since it can clearly depict a network of vascular malformation. In addition, subsequent interventional therapy can be performed immediately after DSA. Follow-up after DSA is necessary due to complications that may occur as a result of hardware manipulation or vascular spasm during the angiographic procedure. In our case, the factors associated with the obliteration of PAVM after DSA are not entirely understood, though the obliteration may be attributed to the low flow dynamics of the small caliber PAVM as well as to the local administration of the contrast agent. We therefore conclude that when PAVM is asymptomatic and diagnosed with non-invasive imaging techniques it should not be evaluated with DSA, in light of the potential risk of the severe complications of acute pancreatitis.

## Abbreviations

CT, computed tomography; DSA, diagnostic angiography; MRI, magnetic resonance imaging; PAVM, pancreatic arteriovenous malformation; TAE, transcatheter arterial embolization; US, ultrasound
